# What sampling device is the most appropriate for vaginal vault cytology in gynaecological cancer follow up?

**DOI:** 10.2478/v10019-012-0019-x

**Published:** 2012-04-11

**Authors:** Del Pup Lino, Canzonieri Vincenzo, Serraino Diego, Campagnutta Elio

**Affiliations:** 1 Gynaecology Oncology Department; 2 Pathology Department and; 3 Epidemiology Unit, National Cancer Institute CRO, Aviano, PN, Italy

**Keywords:** Ayre’s spatula, cytobrush, female cancer, vaginal cytology

## Abstract

**Background:**

In women with cancer-related hysterectomy, the vaginal vault cytology has a low efficacy - when performed by conventional methods – for the early detection of vaginal recurrence. The amount of exfoliated cells collected is generally low because of atrophy, and the vaginal vault corners can be so narrow that the commonly used Ayres spatula cannot often penetrate deeply into them. This prospective study aimed at identifying the advantages obtained in specimens collection using the cytobrush, as compared to the Ayres’s spatula.

**Patients and methods.:**

141 gynaecologic cancer patients were studied to compare samplings collected with Ayre’s spatula or with cytobrush. In a pilot setting of 15 patients, vaginal cytology samples obtained by both Ayre’s spatula and cytobrush were placed at the opposite sites of a single slide for quali-quantitative evaluation. Thereafter, the remaining 126 consecutive women were assigned to either group A (spatula) or B (cytobrush) according to the order of entry. The same gynaecologist performed all the procedures.

**Results:**

In all 15 pilot cases, the cytobrush seemed to collect a higher quantity of material. The comparative analysis of the two complete groups indicated that the cytobrush technique was more effective than the spatula one. The odds ratio (OR) for an optimal cytology using the cytobrush was 2.8 (95% confidence interval -C.I. 1.3–6.2; chi-square test, p=0.008).

**Conclusions:**

Vaginal vault cytology with cytobrush turned out to better perform than the traditional Ayre’s spatula to obtain an adequate sampling in gynecological cancer patients.

## Introduction

The main aim of post-treatment surveillance in oncology is to improve the survival through early detection of recurrent tumours.^1^ The cytopathologic examination is a one of the valuable method to detect an early recurrence of malignancy or new primary carcinoma during the follow-up of patients after the treatment of a different cancer.^2^ However, the vaginal vault cytology is considered a surveillance method with low efficacy for the early detection of vaginal recurrence in patients with a malignancy-related hysterectomy.^3^,^4^ This is partially caused either by cytologic artefacts due to inflammation or by vaginal effects of previous chemotherapy, radiotherapy, or surgery. The amount of exfoliated cells collected is generally low because of atrophy. In addition, the vaginal vault corners can be so narrow that the commonly used Ayres spatula is often unable to penetrate deeply into them. These two latter factors could further reduce the probability of the early detection of a local recurrence, though they can be improved by different sampling methods.

Extended-tip spatulas improve the collection of exfoliated cells, with a nearly 2-fold higher odds ratio (OR) -in comparison with Ayre’s spatula. However, the available data refer to endocervical samples, not to vaginal vault cytology of hysterectomized cancer patients.^5^

To evaluate the best spatula for obtaining adequate vaginal samplings, we reviewed the available literature in PubMed using the following strategy: (“Vaginal Smears/instrumentation”[MeSH] OR “Vaginal Smears/methods”[MeSH] OR “Vaginal Smears/standards”[MeSH] OR “Vaginal Smears/utilization”[MeSH]) AND “Genital Neoplasms, Female”[MeSH] AND “Hysterectomy”[MeSH]. Then we searched for indications on the methods in Plumbed with the strategy: “Follow up” AND “Genital Neoplasms, Female”[Mesh] AND “Hysterectomy”[Mesh] as well as in the main guidelines. We could not find adequate randomized trials regarding the optimal method to perform vaginal vault cytology during the follow-up of hysterectomized women.

## Patients and methods

At the Gynaecology Oncology Department of the National Cancer Institute “Centro di Riferimento Oncologico”, Aviano, North East of Italy, 141 gynaecologic cancer patients in follow-up were included in prospective study. The study design complied with national regulations and institutional policies and the study was carried out according to the Helsinki Declaration.

The survey was conducted in two steps. In a preliminary study of 15 patients, we performed vaginal cytology with both Ayres and cytobrush and the cells samples were placed on the same slide, half of the slide area for each device. In order to prevent bias due to the greater amount of cells collected by the first device used, the first sample was obtained initially with Ayres spatula and subsequently with cytobrush. For every subsequent patient this order was changed. Cytobrush samples were placed on the half of the slide far from patient’s identification data. Criteria of adequacy for vaginal vault cytology consisted in the evaluation of enough cellularity of squamous type. However, since the goal of our work was to identify the best method to collect more vaginal cells for smears, some differences in the amount of cells available for comparisons between the two studied methods were expected. This first step of the study aimed to describe the cytology differences between the two sampling devices analysing the cells on the same slide. The study continued with a methodology that allowed an improved assessment of the differences.

In the second and most important step of the study, 126 consecutive hysterectomized cancer patients who underwent vaginal vault cytology were investigated. Fifty-nine (47%) of them had cervical cancer, 53 (42%) endometrial cancer, nine (8%) had ovarian cancer, four (3%) had vulvar cancer, and one uterine sarcoma. These women were alternatively assigned either to group A (*i.e*., classical Ayres spatula) or B (*i.e*., cytobrush) according to the order of entry, starting from group A. Vaginal vault cytology collection was performed in group A with the classical Ayres spatula and in group B with cytobrush. The same gynaecologist (DPL) performed all the procedures with exactly the same approach. Smears were immediately fixed by cytofix spray with uniform distribution over the smear without artifacts. Staining was conducted by standard Papanicolau method. One pathologist (CV), blinded to the sampling method, analysed all the samples. The cellularity was considered suboptimal when scanty and/or showing an unsuitable morphology for diagnosis. Criteria of adequacy for vaginal vault cytology consisted in the evaluation of adequate cellularity of squamous type. However, since the goal of the work was to establish the best method to collect more vaginal cells for smears, some differences in the amount of cells available for evaluation between the two methods were expected. The Chi-square test for heterogeneity, OR and their 95% CI were computed to assess statistical associations.

## Results

**FIGURE 1:**
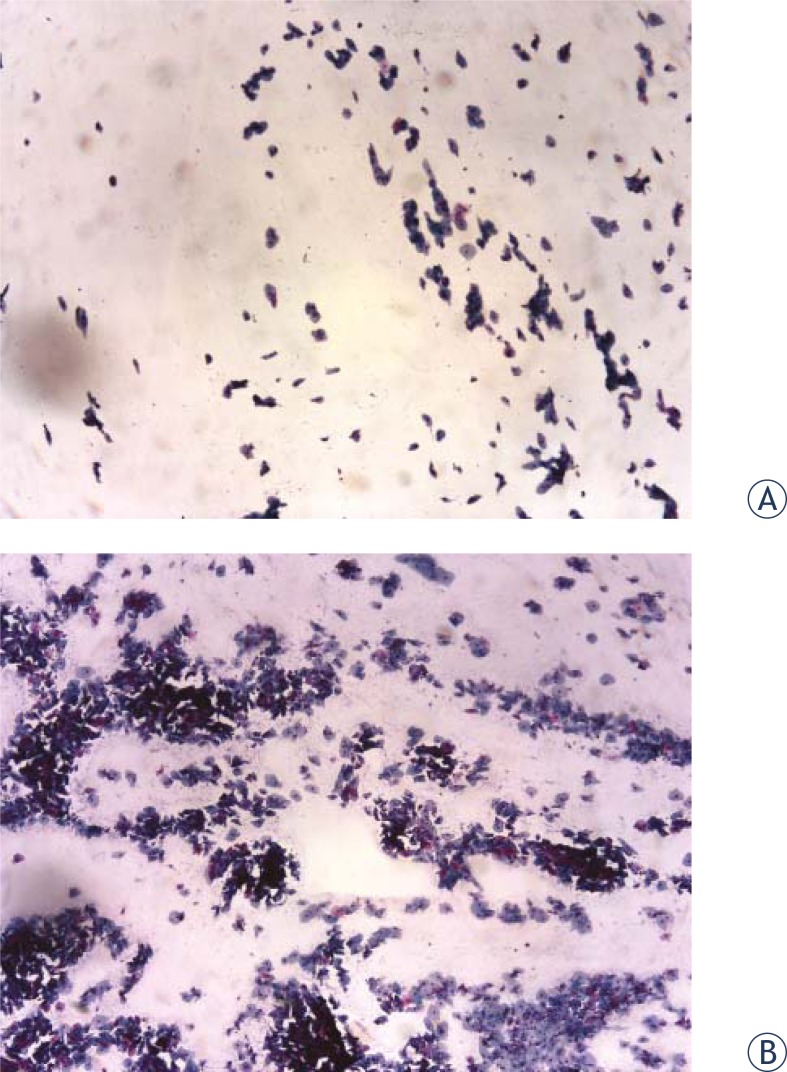
Cytological sample collected from vaginal vault with Ayre’s spatula (A) and cytobrush (B) (Papanicolau; mag. 100X).

All samples were accepted as adequate. In the preliminary study, fifteen specimens showed a higher cellularity using cytobrush (Figure 1). In the second part of the study, among patients where the vaginal cytology with Ayres spatula was performed, 41 patients had a suboptimal quantity of collected cells and 23 of them an optimal one. Among patients who underwent vaginal cytology with cytobrush, 24 had a suboptimal cytology and 38 an optimal one. This difference was statistically significant (p= 0.008), while the OR for an optimal cytology using the cytobrush was 2.8 (95% C.I. 1.3–6.2) (Table 1). There were no side effects, such as bleeding, in both sampling groups.

## Discussion

Our data suggested that the cytobrush is a more efficient sampling device than the traditional Ayre’s spatula btain from vaginal vault. Indeed, it allows collecting 2.8 times the amount of optimal specimen, as it enables more efficient cell scraping from the vaginal epithelium, which is almost invariably atrophic in hysterectomized gynaecologic cancer patients. The cytobrush device carries also the advantage of a deeper sampling by reaching the narrow corners sometimes produced by surgical interventions in the vaginal vault. In these hidden spaces, an initial recurrence can also be difficult to be visualized or palpated. Furthermore, plastic materials tend to have a lower adherence than wooden spatulas, allowing more cells to be smeared to the slide for the analysis.

The Ayre’s spatula is the device commonly used for cervical cytology and the one used in previous vaginal vault cytology studies that are summarized below. Vaginal vault cytology is generally performed during follow-up, after hysterectomy for cervical cancer. Only one patient, out of 79 completing a 15-year follow-up study^6^, had an abnormal smear and a vaginal intraepithelial neoplasia (VAIN) later diagnosed and no patient developed invasive vaginal carcinoma. A French study^7^, conducted over a 10-year period on 2152 patients, reported only four cases of invasive cancer of the vagina of which one occurring after radical hysterectomy for invasive cancer of the cervix and three after total hysterectomy for cervical intraepithelial neoplasia (CIN). The follow-up of endometrial cancer does not seem to improve the survival. A Canadian study^8^ concluded that the routine use of vaginal vault smears was not cost effective during the follow-up. A detection of each asymptomatic vaginal recurrence requires 1067 Pap-smear tests, producing benefits for only 0.5% of patients.^9^ Neither recurrence free nor the overall survival are improved in these cases compared to those detected at clinical presentation.^10^,^11^ Only in non-irradiated patients, a strong case can be made for regular follow-up to detect vaginal recurrence at the earliest opportunity, given the high salvage rate following radiotherapy.^12^,^13^ In a systematic review, the detection of asymptomatic recurrences of endometrial cancer ranged from 0% to 4% with vaginal vault cytology, as compared to 5% to 33% with physical examination.^14^

The effectiveness of vaginal vault cytology in the above mentioned studies could have been different, if performed with the cytobrush. We used both the cytobrush and the Ayre’s spatula for vaginal vault sampling. Smear preparation was made according to standard pathological operative procedures. In our study, we did not use liquid based cytology to increase the whole number of collected cells because the main goal of the study was to compare two sampling methods conducted by means of two different devices.

In conclusion, our data revealed that, in women with gynecological cancers, vaginal vault cytology conducted with the cytobrush appeared more efficient than its conventional counterpart, the Ayre’s spatula, as it allowed the collection of a more adequate sample.

## Figures and Tables

**TABLE 1:** Differences in cytologic adequate sampling (optimal and suboptimal) between the two sampling devices: Ayre’s spatula and cytobrush

		**Optimal**		**Suboptimal**		**Total**
		n	%	n	%	n
Group A:	Ayre’s spatula	23	35.9	41	64.1	64
Group B:	Cytobrush	38	61.3	24	38.7	62
		65		61		126

(Chi square test, p= 0.008)
